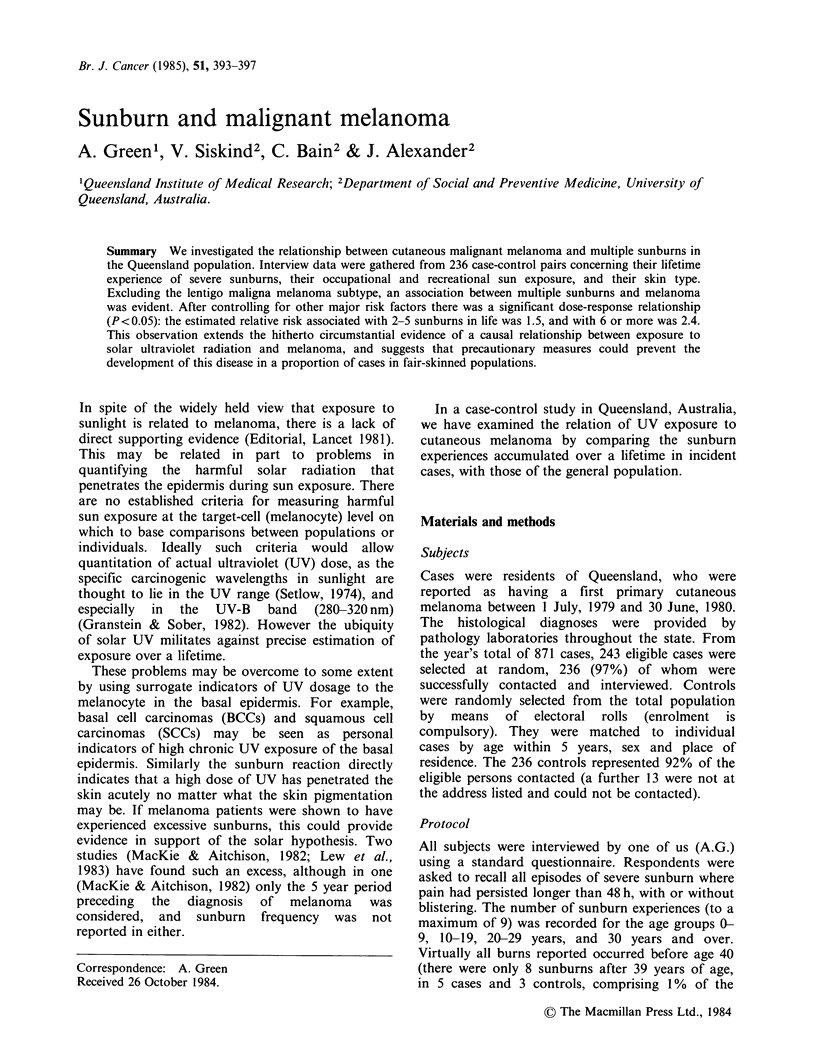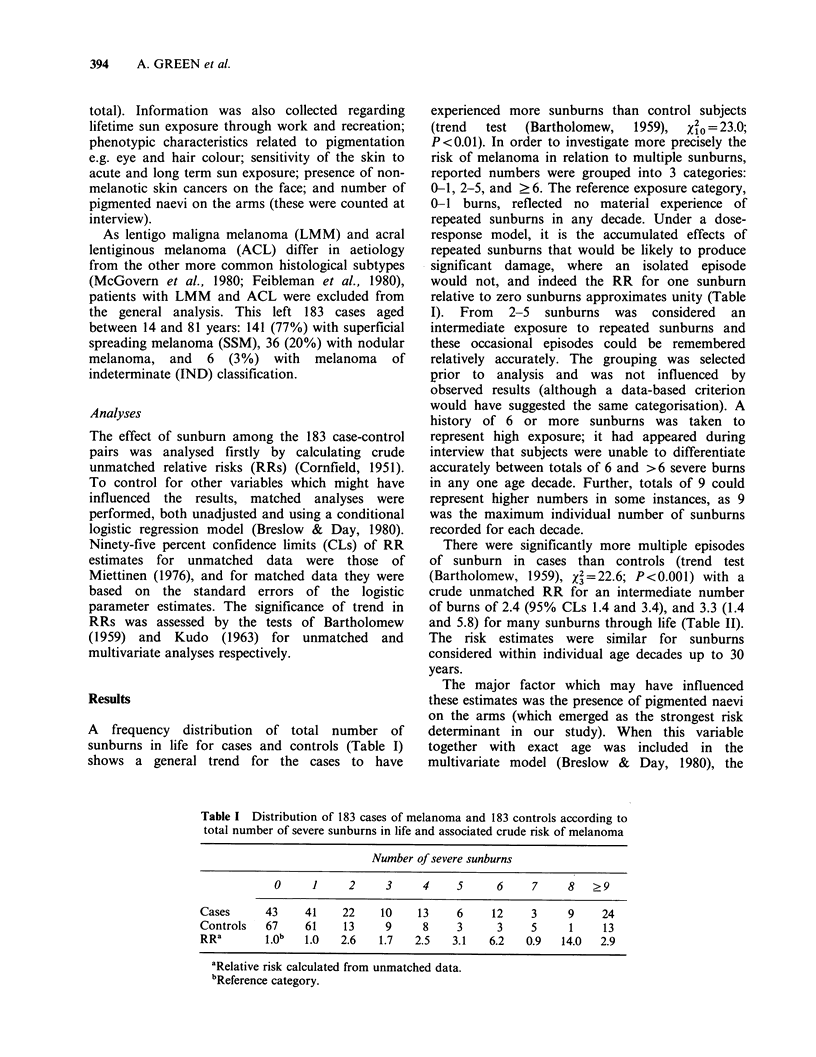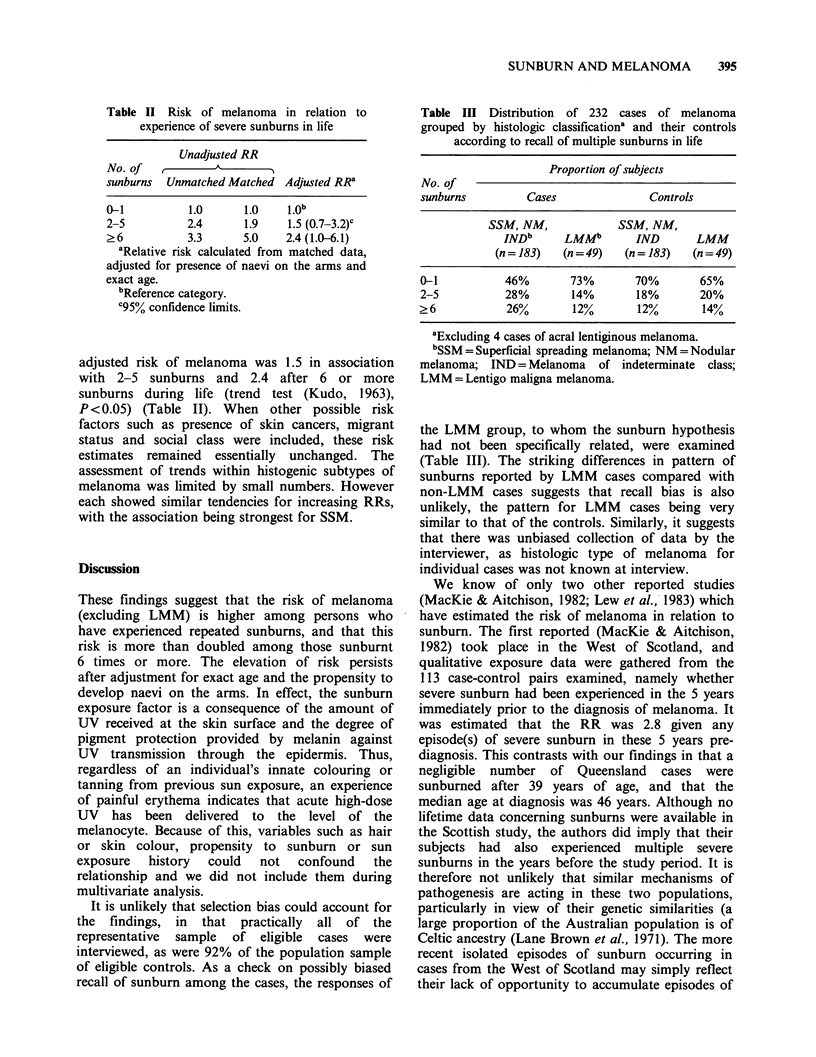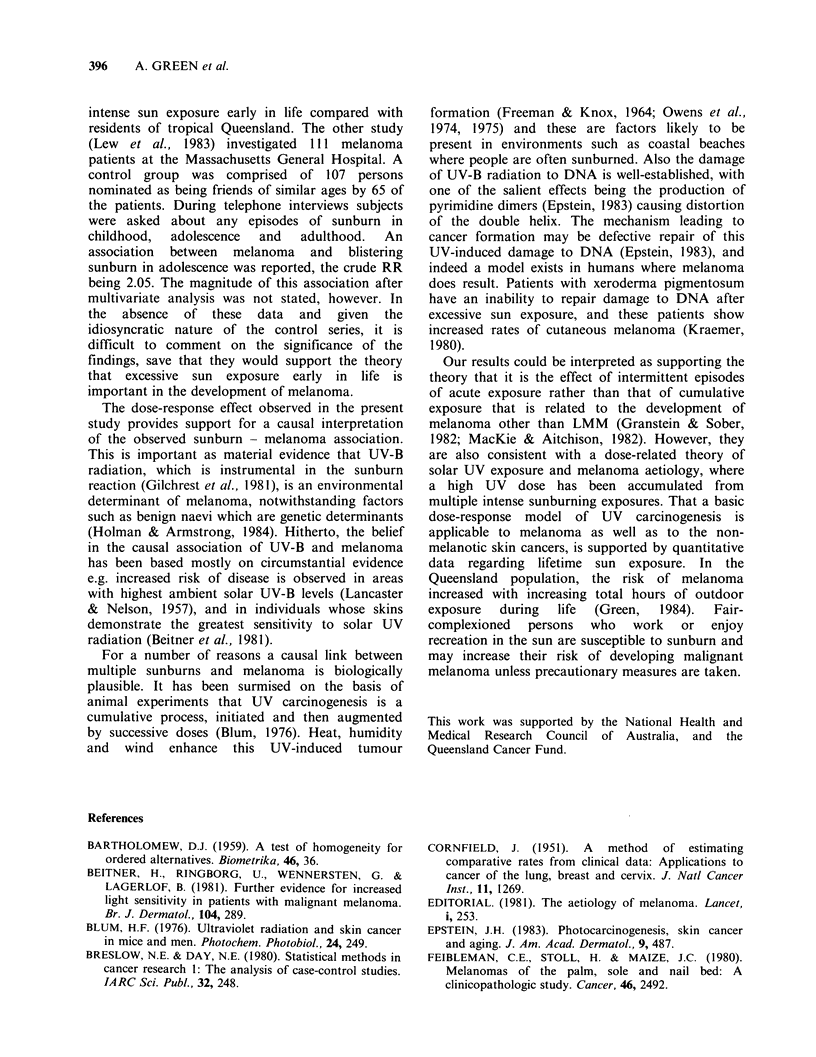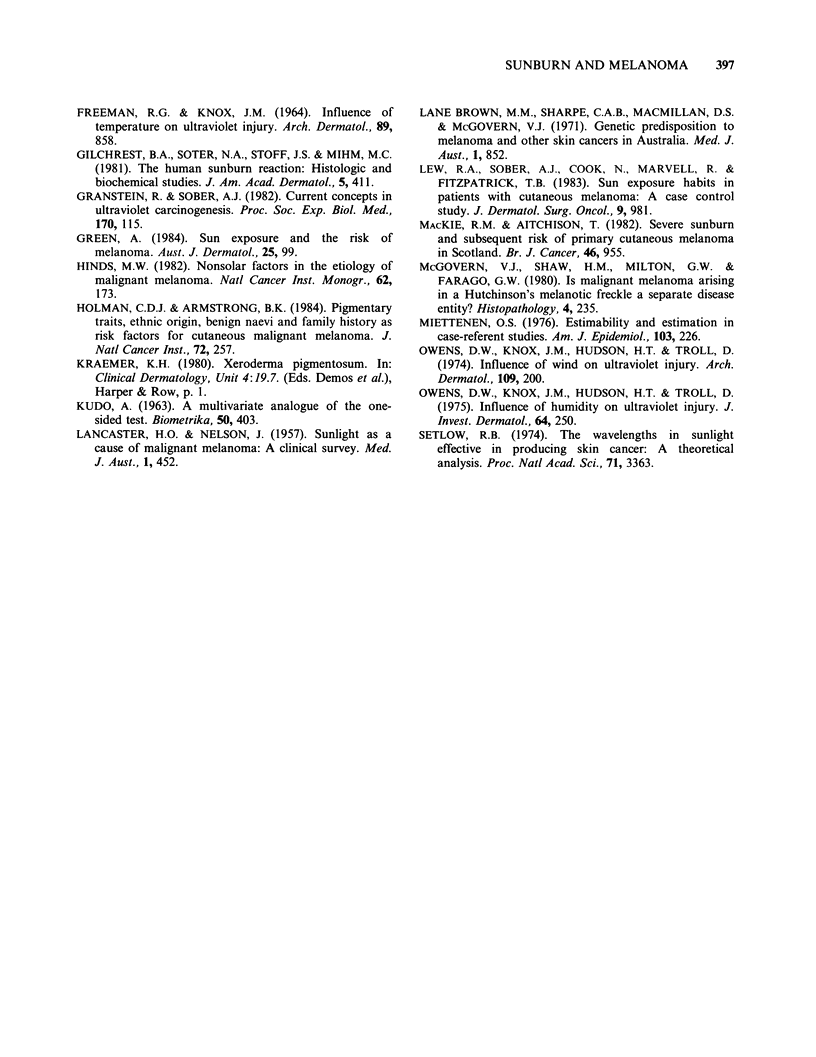# Sunburn and malignant melanoma.

**DOI:** 10.1038/bjc.1985.53

**Published:** 1985-03

**Authors:** A. Green, V. Siskind, C. Bain, J. Alexander

## Abstract

We investigated the relationship between cutaneous malignant melanoma and multiple sunburns in the Queensland population. Interview data were gathered from 236 case-control pairs concerning their lifetime experience of severe sunburns, their occupational and recreational sun exposure, and their skin type. Excluding the lentigo maligna melanoma subtype, an association between multiple sunburns and melanoma was evident. After controlling for other major risk factors there was a significant dose-response relationship (P less than 0.05): the estimated relative risk associated with 2-5 sunburns in life was 1.5, and with 6 or more was 2.4. This observation extends the hitherto circumstantial evidence of a causal relationship between exposure to solar ultraviolet radiation and melanoma, and suggests that precautionary measures could prevent the development of this disease in a proportion of cases in fair-skinned populations.


*Br. J. Cancer* (1985), **51**, 393–397

### Sunburn and malignant melanoma

A. Green[1], V. Siskind[2], C. Bain[2] & J. Alexander[2]

[1]*Queensland Institute of Medical Research;* [2]*Department of Social and Preventive Medicine, University of Queensland, Australia.*

**Summary** We investigated the relationship between cutaneous malignant melanoma and multiple sunburns in the Queensland population. Interview data were gathered from 236 case-control pairs concerning their lifetime experience of severe sunburns, their occupational and recreational sun exposure, and their skin type. Excluding the lentigo maligna melanoma subtype, an association between multiple sunburns and melanoma was evident. After controlling for other major risk factors there was a significant dose-response relationship ($P<0.05$): the estimated relative risk associated with 2–5 sunburns in life was 1.5, and with 6 or more was 2.4. This observation extends the hitherto circumstantial evidence of a causal relationship between exposure to solar ultraviolet radiation and melanoma, and suggests that precautionary measures could prevent the development of this disease in a proportion of cases in fair-skinned populations.

In spite of the widely held view that exposure to sunlight is related to melanoma, there is a lack of direct supporting evidence (Editorial, Lancet 1981). This may be related in part to problems in quantifying the harmful solar radiation that penetrates the epidermis during sun exposure. There are no established criteria for measuring harmful sun exposure at the target-cell (melanocyte) level on which to base comparisons between populations or individuals. Ideally such criteria would allow quantitation of actual ultraviolet (UV) dose, as the specific carcinogenic wavelengths in sunlight are thought to lie in the UV range (Setlow, 1974), and especially in the UV-B band (280–320 nm) (Granstein & Sober, 1982). However the ubiquity of solar UV militates against precise estimation of exposure over a lifetime.

These problems may be overcome to some extent by using surrogate indicators of UV dosage to the melanocyte in the basal epidermis. For example, basal cell carcinomas (BCCs) and squamous cell carcinomas (SCCs) may be seen as personal indicators of high chronic UV exposure of the basal epidermis. Similarly the sunburn reaction directly indicates that a high dose of UV has penetrated the skin acutely no matter what the skin pigmentation may be. If melanoma patients were shown to have experienced excessive sunburns, this could provide evidence in support of the solar hypothesis. Two studies (MacKie & Aitchison, 1982; Lew *et al.*, 1983) have found such an excess, although in one (MacKie & Aitchison, 1982) only the 5 year period preceding the diagnosis of melanoma was considered, and sunburn frequency was not reported in either.

Correspondence: A. Green
Received 26 October 1984.

In a case-control study in Queensland, Australia, we have examined the relation of UV exposure to cutaneous melanoma by comparing the sunburn experiences accumulated over a lifetime in incident cases, with those of the general population.

### Materials and methods

### *Subjects*

Cases were residents of Queensland, who were reported as having a first primary cutaneous melanoma between 1 July, 1979 and 30 June, 1980. The histological diagnoses were provided by pathology laboratories throughout the state. From the year's total of 871 cases, 243 eligible cases were selected at random, 236 (97%) of whom were successfully contacted and interviewed. Controls were randomly selected from the total population by means of electoral rolls (enrolment is compulsory). They were matched to individual cases by age within 5 years, sex and place of residence. The 236 controls represented 92% of the eligible persons contacted (a further 13 were not at the address listed and could not be contacted).

### *Protocol*

All subjects were interviewed by one of us (A.G.) using a standard questionnaire. Respondents were asked to recall all episodes of severe sunburn where pain had persisted longer than 48 h, with or without blistering. The number of sunburn experiences (to a maximum of 9) was recorded for the age groups 0–9, 10–19, 20–29 years, and 30 years and over. Virtually all burns reported occurred before age 40 (there were only 8 sunburns after 39 years of age, in 5 cases and 3 controls, comprising 1% of the

© The Macmillan Press Ltd., 1984

total). Information was also collected regarding lifetime sun exposure through work and recreation; phenotypic characteristics related to pigmentation e.g. eye and hair colour; sensitivity of the skin to acute and long term sun exposure; presence of non-melanotic skin cancers on the face; and number of pigmented naevi on the arms (these were counted at interview).

As lentigo maligna melanoma (LMM) and acral lentiginous melanoma (ACL) differ in aetiology from the other more common histological subtypes (McGovern *et al.*, 1980; Feibleman *et al.*, 1980), patients with LMM and ACL were excluded from the general analysis. This left 183 cases aged between 14 and 81 years: 141 (77%) with superficial spreading melanoma (SSM), 36 (20%) with nodular melanoma, and 6 (3%) with melanoma of indeterminate (IND) classification.

### *Analyses*

The effect of sunburn among the 183 case-control pairs was analysed firstly by calculating crude unmatched relative risks (RRs) (Cornfield, 1951). To control for other variables which might have influenced the results, matched analyses were performed, both unadjusted and using a conditional logistic regression model (Breslow & Day, 1980). Ninety-five percent confidence limits (CLs) of RR estimates for unmatched data were those of Miettinen (1976), and for matched data they were based on the standard errors of the logistic parameter estimates. The significance of trend in RRs was assessed by the tests of Bartholomew (1959) and Kudo (1963) for unmatched and multivariate analyses respectively.

### Results

A frequency distribution of total number of sunburns in life for cases and controls (Table I) shows a general trend for the cases to have experienced more sunburns than control subjects (trend test (Bartholomew, 1959), $\chi^2_{10}=23.0$; $P<0.01$). In order to investigate more precisely the risk of melanoma in relation to multiple sunburns, reported numbers were grouped into 3 categories: 0–1, 2–5, and $\geq 6$. The reference exposure category, 0–1 burns, reflected no material experience of repeated sunburns in any decade. Under a dose-response model, it is the accumulated effects of repeated sunburns that would be likely to produce significant damage, where an isolated episode would not, and indeed the RR for one sunburn relative to zero sunburns approximates unity (Table I). From 2–5 sunburns was considered an intermediate exposure to repeated sunburns and these occasional episodes could be remembered relatively accurately. The grouping was selected prior to analysis and was not influenced by observed results (although a data-based criterion would have suggested the same categorisation). A history of 6 or more sunburns was taken to represent high exposure; it had appeared during interview that subjects were unable to differentiate accurately between totals of 6 and $>6$ severe burns in any one age decade. Further, totals of 9 could represent higher numbers in some instances, as 9 was the maximum individual number of sunburns recorded for each decade.

There were significantly more multiple episodes of sunburn in cases than controls (trend test (Bartholomew, 1959), $\chi^2_3=22.6$; $P<0.001$) with a crude unmatched RR for an intermediate number of burns of 2.4 (95% CLs 1.4 and 3.4), and 3.3 (1.4 and 5.8) for many sunburns through life (Table II). The risk estimates were similar for sunburns considered within individual age decades up to 30 years.

The major factor which may have influenced these estimates was the presence of pigmented naevi on the arms (which emerged as the strongest risk determinant in our study). When this variable together with exact age was included in the multivariate model (Breslow & Day, 1980), the

**Table I** Distribution of 183 cases of melanoma and 183 controls according to total number of severe sunburns in life and associated crude risk of melanoma

| | *Number of severe sunburns* | | | | | | | | | |
|---|---|---|---|---|---|---|---|---|---|---|
| | *0* | *1* | *2* | *3* | *4* | *5* | *6* | *7* | *8* | *≥9* |
| Cases | 43 | 41 | 22 | 10 | 13 | 6 | 12 | 3 | 9 | 24 |
| Controls | 67 | 61 | 13 | 9 | 8 | 3 | 3 | 5 | 1 | 13 |
| RR[a] | 1.0[b] | 1.0 | 2.6 | 1.7 | 2.5 | 3.1 | 6.2 | 0.9 | 14.0 | 2.9 |

[a]Relative risk calculated from unmatched data.
[b]Reference category.

**Table II** Risk of melanoma in relation to experience of severe sunburns in life

| No. of sunburns | Unadjusted RR: Unmatched | Unadjusted RR: Matched | Adjusted RR[a] |
|---|---|---|---|
| 0–1 | 1.0 | 1.0 | 1.0[b] |
| 2–5 | 2.4 | 1.9 | 1.5 (0.7–3.2)[c] |
| ≥6 | 3.3 | 5.0 | 2.4 (1.0–6.1) |

[a]Relative risk calculated from matched data, adjusted for presence of naevi on the arms and exact age.
[b]Reference category.
[c]95% confidence limits.

**Table III** Distribution of 232 cases of melanoma grouped by histologic classification[a] and their controls according to recall of multiple sunburns in life

| No. of sunburns | Proportion of subjects: Cases | | Controls | |
|---|---|---|---|---|
| | SSM, NM, IND[b] (n=183) | LMM[b] (n=49) | SSM, NM, IND (n=183) | LMM (n=49) |
| 0–1 | 46% | 73% | 70% | 65% |
| 2–5 | 28% | 14% | 18% | 20% |
| ≥6 | 26% | 12% | 12% | 14% |

[a]Excluding 4 cases of acral lentiginous melanoma.
[b]SSM = Superficial spreading melanoma; NM = Nodular melanoma; IND = Melanoma of indeterminate class; LMM = Lentigo maligna melanoma.

adjusted risk of melanoma was 1.5 in association with 2–5 sunburns and 2.4 after 6 or more sunburns during life (trend test (Kudo, 1963), $P<0.05$) (Table II). When other possible risk factors such as presence of skin cancers, migrant status and social class were included, these risk estimates remained essentially unchanged. The assessment of trends within histogenic subtypes of melanoma was limited by small numbers. However each showed similar tendencies for increasing RRs, with the association being strongest for SSM.

### Discussion

These findings suggest that the risk of melanoma (excluding LMM) is higher among persons who have experienced repeated sunburns, and that this risk is more than doubled among those sunburnt 6 times or more. The elevation of risk persists after adjustment for exact age and the propensity to develop naevi on the arms. In effect, the sunburn exposure factor is a consequence of the amount of UV received at the skin surface and the degree of pigment protection provided by melanin against UV transmission through the epidermis. Thus, regardless of an individual's innate colouring or tanning from previous sun exposure, an experience of painful erythema indicates that acute high-dose UV has been delivered to the level of the melanocyte. Because of this, variables such as hair or skin colour, propensity to sunburn or sun exposure history could not confound the relationship and we did not include them during multivariate analysis.

It is unlikely that selection bias could account for the findings, in that practically all of the representative sample of eligible cases were interviewed, as were 92% of the population sample of eligible controls. As a check on possibly biased recall of sunburn among the cases, the responses of the LMM group, to whom the sunburn hypothesis had not been specifically related, were examined (Table III). The striking differences in pattern of sunburns reported by LMM cases compared with non-LMM cases suggests that recall bias is also unlikely, the pattern for LMM cases being very similar to that of the controls. Similarly, it suggests that there was unbiased collection of data by the interviewer, as histologic type of melanoma for individual cases was not known at interview.

We know of only two other reported studies (MacKie & Aitchison, 1982; Lew *et al.*, 1983) which have estimated the risk of melanoma in relation to sunburn. The first reported (MacKie & Aitchison, 1982) took place in the West of Scotland, and qualitative exposure data were gathered from the 113 case-control pairs examined, namely whether severe sunburn had been experienced in the 5 years immediately prior to the diagnosis of melanoma. It was estimated that the RR was 2.8 given any episode(s) of severe sunburn in these 5 years pre-diagnosis. This contrasts with our findings in that a negligible number of Queensland cases were sunburned after 39 years of age, and that the median age at diagnosis was 46 years. Although no lifetime data concerning sunburns were available in the Scottish study, the authors did imply that their subjects had also experienced multiple severe sunburns in the years before the study period. It is therefore not unlikely that similar mechanisms of pathogenesis are acting in these two populations, particularly in view of their genetic similarities (a large proportion of the Australian population is of Celtic ancestry (Lane Brown *et al.*, 1971). The more recent isolated episodes of sunburn occurring in cases from the West of Scotland may simply reflect their lack of opportunity to accumulate episodes of intense sun exposure early in life compared with residents of tropical Queensland. The other study (Lew *et al.*, 1983) investigated 111 melanoma patients at the Massachusetts General Hospital. A control group was comprised of 107 persons nominated as being friends of similar ages by 65 of the patients. During telephone interviews subjects were asked about any episodes of sunburn in childhood, adolescence and adulthood. An association between melanoma and blistering sunburn in adolescence was reported, the crude RR being 2.05. The magnitude of this association after multivariate analysis was not stated, however. In the absence of these data and given the idiosyncratic nature of the control series, it is difficult to comment on the significance of the findings, save that they would support the theory that excessive sun exposure early in life is important in the development of melanoma.

The dose-response effect observed in the present study provides support for a causal interpretation of the observed sunburn – melanoma association. This is important as material evidence that UV-B radiation, which is instrumental in the sunburn reaction (Gilchrest *et al.*, 1981), is an environmental determinant of melanoma, notwithstanding factors such as benign naevi which are genetic determinants (Holman & Armstrong, 1984). Hitherto, the belief in the causal association of UV-B and melanoma has been based mostly on circumstantial evidence e.g. increased risk of disease is observed in areas with highest ambient solar UV-B levels (Lancaster & Nelson, 1957), and in individuals whose skins demonstrate the greatest sensitivity to solar UV radiation (Beitner *et al.*, 1981).

For a number of reasons a causal link between multiple sunburns and melanoma is biologically plausible. It has been surmised on the basis of animal experiments that UV carcinogenesis is a cumulative process, initiated and then augmented by successive doses (Blum, 1976). Heat, humidity and wind enhance this UV-induced tumour formation (Freeman & Knox, 1964; Owens *et al.*, 1974, 1975) and these are factors likely to be present in environments such as coastal beaches where people are often sunburned. Also the damage of UV-B radiation to DNA is well-established, with one of the salient effects being the production of pyrimidine dimers (Epstein, 1983) causing distortion of the double helix. The mechanism leading to cancer formation may be defective repair of this UV-induced damage to DNA (Epstein, 1983), and indeed a model exists in humans where melanoma does result. Patients with xeroderma pigmentosum have an inability to repair damage to DNA after excessive sun exposure, and these patients show increased rates of cutaneous melanoma (Kraemer, 1980).

Our results could be interpreted as supporting the theory that it is the effect of intermittent episodes of acute exposure rather than that of cumulative exposure that is related to the development of melanoma other than LMM (Granstein & Sober, 1982; MacKie & Aitchison, 1982). However, they are also consistent with a dose-related theory of solar UV exposure and melanoma aetiology, where a high UV dose has been accumulated from multiple intense sunburning exposures. That a basic dose-response model of UV carcinogenesis is applicable to melanoma as well as to the non-melanotic skin cancers, is supported by quantitative data regarding lifetime sun exposure. In the Queensland population, the risk of melanoma increased with increasing total hours of outdoor exposure during life (Green, 1984). Fair-complexioned persons who work or enjoy recreation in the sun are susceptible to sunburn and may increase their risk of developing malignant melanoma unless precautionary measures are taken.

This work was supported by the National Health and Medical Research Council of Australia, and the Queensland Cancer Fund.

### References

BARTHOLOMEW, D.J. (1959). A test of homogeneity for ordered alternatives. *Biometrika*, **46**, 36.

BEITNER, H., RINGBORG, U., WENNERSTEN, G. & LAGERLOF, B. (1981). Further evidence for increased light sensitivity in patients with malignant melanoma. *Br. J. Dermatol.*, **104**, 289.

BLUM, H.F. (1976). Ultraviolet radiation and skin cancer in mice and men. *Photochem. Photobiol.*, **24**, 249.

BRESLOW, N.E. & DAY, N.E. (1980). Statistical methods in cancer research 1: The analysis of case-control studies. *IARC Sci. Publ.*, **32**, 248.

CORNFIELD, J. (1951). A method of estimating comparative rates from clinical data: Applications to cancer of the lung, breast and cervix. *J. Natl Cancer Inst.*, **11**, 1269.

EDITORIAL. (1981). The aetiology of melanoma. *Lancet*, **i**, 253.

EPSTEIN, J.H. (1983). Photocarcinogenesis, skin cancer and aging. *J. Am. Acad. Dermatol.*, **9**, 487.

FEIBLEMAN, C.E., STOLL, H. & MAIZE, J.C. (1980). Melanomas of the palm, sole and nail bed: A clinicopathologic study. *Cancer*, **46**, 2492.

FREEMAN, R.G. & KNOX, J.M. (1964). Influence of temperature on ultraviolet injury. *Arch. Dermatol.*, **89**, 858.

GILCHREST, B.A., SOTER, N.A., STOFF, J.S. & MIHM, M.C. (1981). The human sunburn reaction: Histologic and biochemical studies. *J. Am. Acad. Dermatol.*, **5**, 411.

GRANSTEIN, R. & SOBER, A.J. (1982). Current concepts in ultraviolet carcinogenesis. *Proc. Soc. Exp. Biol. Med.*, **170**, 115.

GREEN, A. (1984). Sun exposure and the risk of melanoma. *Aust. J. Dermatol.*, **25**, 99.

HINDS, M.W. (1982). Nonsolar factors in the etiology of malignant melanoma. *Natl Cancer Inst. Monogr.*, **62**, 173.

HOLMAN, C.D.J. & ARMSTRONG, B.K. (1984). Pigmentary traits, ethnic origin, benign naevi and family history as risk factors for cutaneous malignant melanoma. *J. Natl Cancer Inst.*, **72**, 257.

KRAEMER, K.H. (1980). Xeroderma pigmentosum. In: *Clinical Dermatology, Unit 4:19.7.* (Eds. Demos *et al.*), Harper & Row, p. 1.

KUDO, A. (1963). A multivariate analogue of the one-sided test. *Biometrika*, **50**, 403.

LANCASTER, H.O. & NELSON, J. (1957). Sunlight as a cause of malignant melanoma: A clinical survey. *Med. J. Aust.*, **1**, 452.

LANE BROWN, M.M., SHARPE, C.A.B., MACMILLAN, D.S. & McGOVERN, V.J. (1971). Genetic predisposition to melanoma and other skin cancers in Australia. *Med. J. Aust.*, **1**, 852.

LEW, R.A., SOBER, A.J., COOK, N., MARVELL, R. & FITZPATRICK, T.B. (1983). Sun exposure habits in patients with cutaneous melanoma: A case control study. *J. Dermatol. Surg. Oncol.*, **9**, 981.

MacKIE, R.M. & AITCHISON, T. (1982). Severe sunburn and subsequent risk of primary cutaneous melanoma in Scotland. *Br. J. Cancer*, **46**, 955.

McGOVERN, V.J., SHAW, H.M., MILTON, G.W. & FARAGO, G.W. (1980). Is malignant melanoma arising in a Hutchinson's melanotic freckle a separate disease entity? *Histopathology*, **4**, 235.

MIETTENEN, O.S. (1976). Estimability and estimation in case-referent studies. *Am. J. Epidemiol.*, **103**, 226.

OWENS, D.W., KNOX, J.M., HUDSON, H.T. & TROLL, D. (1974). Influence of wind on ultraviolet injury. *Arch. Dermatol.*, **109**, 200.

OWENS, D.W., KNOX, J.M., HUDSON, H.T. & TROLL, D. (1975). Influence of humidity on ultraviolet injury. *J. Invest. Dermatol.*, **64**, 250.

SETLOW, R.B. (1974). The wavelengths in sunlight effective in producing skin cancer: A theoretical analysis. *Proc. Natl Acad. Sci.*, **71**, 3363.